# PyPropel: a Python-based tool for efficiently processing and characterising protein data

**DOI:** 10.1186/s12859-025-06079-3

**Published:** 2025-03-01

**Authors:** Jianfeng Sun, Jinlong Ru, Adam P. Cribbs, Dapeng Xiong

**Affiliations:** 1 Botnar Research Centre, University of Oxford, Headington, Oxford, OX3 7LD UK; 2 Chair of Prevention of Microbial Diseases, School of Life Sciences Weihenstephan, Technical University of Munich, 85354 Freising, Germany; 3 Department of Computational Biology, Cornell University, Ithaca, 14853 USA; 4 Weill Institute for Cell and Molecular Biology, Cornell University, Ithaca, 14853 USA

**Keywords:** Sequence analysis, Protein features, Data pre-processing, Structural bioinformatics, Machine learning

## Abstract

**Background:**

The volume of protein sequence data has grown exponentially in recent years, driven by advancements in metagenomics. Despite this, a substantial proportion of these sequences remain poorly annotated, underscoring the need for robust bioinformatics tools to facilitate efficient characterisation and annotation for functional studies.

**Results:**

We present PyPropel, a Python-based computational tool developed to streamline the large-scale analysis of protein data, with a particular focus on applications in machine learning. PyPropel integrates sequence and structural data pre-processing, feature generation, and post-processing for model performance evaluation and visualisation, offering a comprehensive solution for handling complex protein datasets.

**Conclusion:**

PyPropel provides added value over existing tools by offering a unified workflow that encompasses the full spectrum of protein research, from raw data pre-processing to functional annotation and model performance analysis, thereby supporting efficient protein function studies.

**Supplementary Information:**

The online version contains supplementary material available at 10.1186/s12859-025-06079-3.

## Introduction

Advanced sequencing technologies have significantly accelerated the discovery of genomic and transcriptomic sequences, leading to a substantial expansion of the known protein space [1]. Metagenomic approaches have been the primary contributors to this growth [2], with microbial protein sequences increasing by approximately 50% annually, as reported by the UniProt database [3]. Despite this surge, as of December 2024, less than 0.3% of the sequences deposited in UniProt (572,619 reviewed vs. 253,682,368 unreviewed) have been manually annotated [3]. The key challenge remains the development of bioinformatics tools capable of efficiently characterising and annotating these protein sequences at both the sequence- and site-specific levels to facilitate functional studies [4].

To facilitate the functional characterisation of protein sequences, various computational tools have been devised to query established biological databases for known properties (e.g., amino acid physiochemical properties [5]) or to predict unknown attributes (e.g., protein–protein interaction sites [6] and variant effects [7]). Most of these tools focus on feature generation for a given set of protein sequences [8–12]. However, the datasets used are often pre-processed and generated independently from large-scale protein databases by separate tools [13] or custom scripts, resulting in a time-consuming workflow. Moreover, in the post-processing stage, integrating diverse protein features from multiple tools for machine learning and performance evaluation can be challenging. Currently, there are few tools that provide a comprehensive solution covering both pre- and post-processing stages of protein sequence analysis.

In this work, we describe PyPropel, a Python package designed to streamline the handling of protein sequence data, with a focus on machine learning applications. PyPropel provides a wide range of functionalities to facilitate the pre-processing, structural and functional depiction, and post-processing of protein sequence data. In the pre-processing stage, it enables users to retrieve sequence and structural data while enhancing the quality of custom-built datasets, such as converting between interchangeable formats for multiple sequence alignments (MSAs) [14]. PyPropel can also query UniProt for structural and functional information given protein entries (e.g., experimental evidence of binding sites) or generate annotations by reprocessing results from built-in functions or external tools (e.g., relative solvent accessibility [15]). Additionally, PyPropel supports the integration of protein features from multiple sources and allows for performance comparison in tasks involving single amino acid predictions (e.g. disordered sites [16]). By refining the processing workflow for protein sequencing data, PyPropel complements existing bioinformatics tools and enhances protein functional research.

### Implementation

PyPropel is designed modular and scalable, offering bioinformatics researchers seamless integration with external computing libraries and tools. For instance, protein entries screened by other tools (e.g., TMKit [17], a tool we previously developed for transmembrane proteins) can be effortlessly passed into PyPropel for sequence and structural data generation and quality control within a Jupyter notebook or a Python script. Protein features are organised in a two-dimensional Python list, allowing for easy inclusion or exclusion of specific features as required. This flexible architecture supports the training of machine learning models under various criteria, simplifying the process of comparing results with baseline models.

## Results

### Overview of PyPropel

**Fig. 1:**
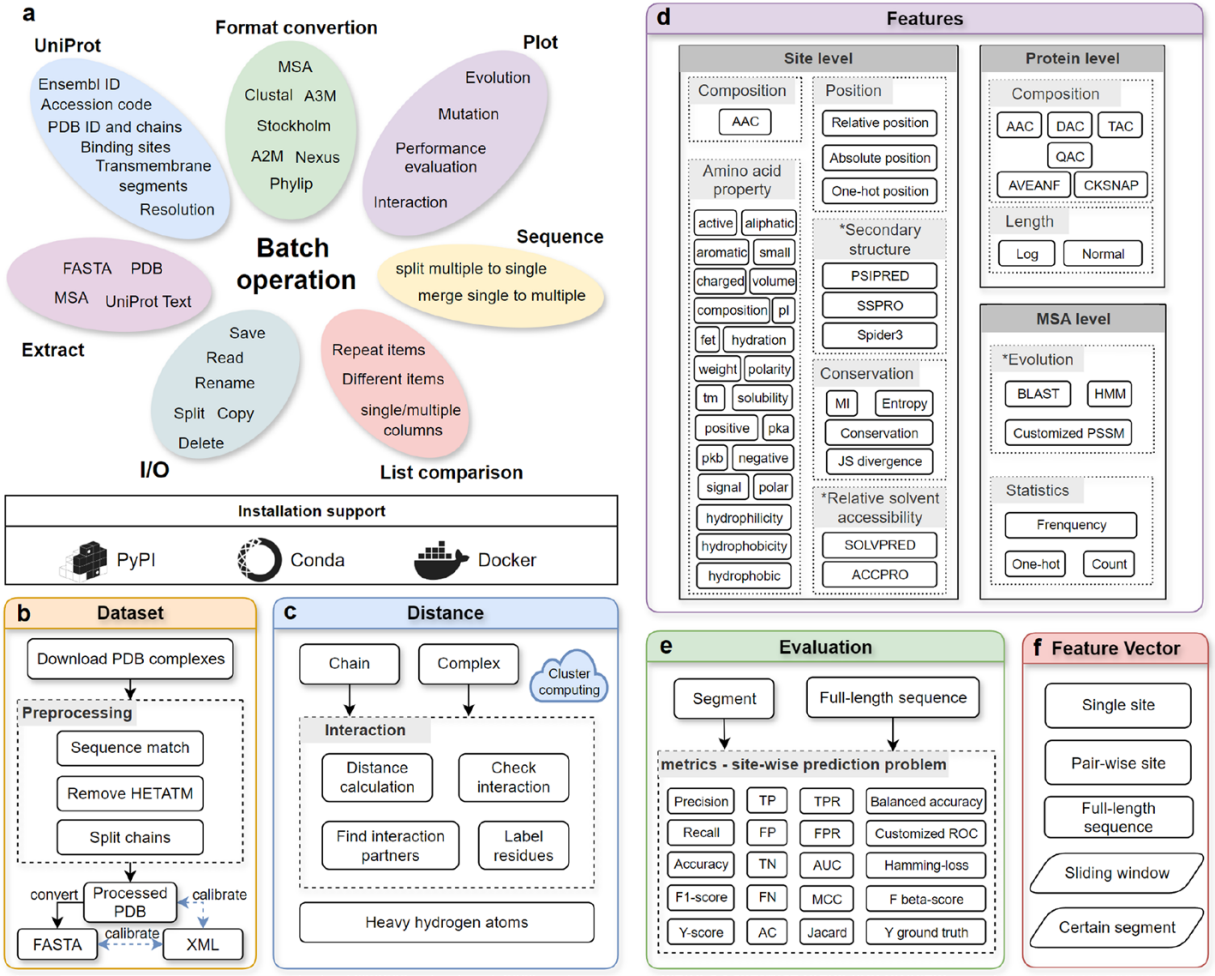
Overview of main characteristics of PyPropel, including (**a**) batch operation of data, (**b**) dataset preprocessing, (**c**) distance calculation, (**d**) feature generation, (**e**) performance evaluation, and (**f**) feature vector support. **a**. PyPropel functions robustly in file I/O operation and batch-processes multiple kinds of files. **b**. PyPropel can automate the generation of datasets once a list of molecular identifiers is supplied. **c**. It contains functions to calculate distance between amino acid residues to label samples for structural bioinformatics. This is an important step to generate ground-truth labels for machine learning. **d**. At the core of PyPropel is the module for feature generation, which helps the structural and functional characterisation of protein sequences, particularly facilitating sequence-based prediction problem studies. Once labels and features are generated, the data can directly be passed on to machine learning libraries (e.g. scikit-learn, with two examples demonstrated on PyPropel’s documentation) to train models. Finally, PyPropel supports the calculation of evaluation metrics of model performance. **f**. Note that PyPropel supplies various vectors to accommodate the features of individual residues, entire protein sequences, or residue pairs. PyPropel is a cross-platform tool implemented with Python. The asterisk (*) represents the reliance of feature generation on external files

PyPropel offers a suite of functions for pre-processing, characterising, and post-processing protein sequence, structural, and functional data (Fig. 1). When used in conjunction with TMKit, PyPropel provides a comprehensive workflow for preparing and generating protein datasets and feature sets required for machine learning studies.

### Pre-processing sequence and structural data

#### Retrieval and quality control of sequence and structural data.

PyPropel automates the bulk retrieval of topological records in XML formats [18, 19] and Protein Data Bank (PDB) structures [20] based on a list of protein entries, followed by comprehensive quality control. During this process, the structural data is assessed to ensure exclusion of HETATM atoms and the absence of unknown amino acids (i.e., annotated as UNK in PDB files). PyPropel also facilitates the seamless extraction and conversion of sequences across multiple formats, ensuring accurate residue numbering and consistent tracking. Additionally, PyPropel optimises the computationally intensive task of identifying discrepancies between large protein lists, enabling the comparison of tens of millions of protein identifiers to be completed within seconds.

#### Distances between residues at intra- and inter-protein levels

Calculating residue distances within or between protein chains and complexes is crucial for identifying functionally and structurally significant residues [21]. In the absence of experimental evidence for functional sites, residues that are spatially proximal at the inter-protein level are often inferred to be potential interaction sites [6, 22]. By applying a distance cutoff, residues can be categorised as interacting or non-interacting. Distance measurements can be made using heavy atoms (i.e., non-hydrogen) [23] or ${C}_{\alpha }$ atoms [24] allowing for precise identification of protein–protein interactions within complexes. This facilitates the generation of a list of interaction sites and their respective distances.

### Characterisation of protein sequences

#### Intrinsic protein features

PyPropel provides various features that capture multiple aspects of protein sequences, including compositional, conservation, length, positional, profile, relative solvent accessibility (RSA), physicochemical, and secondary structure properties. These features are applicable to entire protein sequences, multiple sequence alignments (MSAs), and individual amino acid sites. Some, such as 23 physicochemical properties derived from existing literature [5, 25–28], are permanently integrated within PyPropel, while others, like RSA, are dynamically calculated using outputs from external tools. This extensive range of features enables detailed analysis and modelling of protein characteristics, supporting a broad spectrum of functional and structural studies.

#### Extract experimental evidence using UniProt databases

UniProt is a comprehensive repository of protein sequences, as well as structural and functional annotations, providing data on transmembrane segments, multiple protein identifier versions, experimentally resolved structures, and binding sites. However, bulk access to this information via the webserver or public API can be inefficient. To overcome this limitation, we developed a module within PyPropel that facilitates the rapid extraction of UniProt data, either at the species level or for customized proteins sets, significantly streamlining the retrieval process for large-scale analyses.

### Post-processing sequence and structural data

#### Feature extraction

Protein features in PyPropel are derived from various sources and are organised in a 2D list, allowing for flexible inclusion or exclusion of different feature categories. Features can be assigned to single or pairwise sites within specific regions, such as transmembrane segments of interests (Table 1). Additionally, a sliding window centred at each selected site can be applied to characterise the surrounding sequence context. The effectiveness of this approach has been demonstrated for assigning features to residues for site interaction prediction [29].

**Table 1:** Summary of assembling features using feature vectors for prediction problems

Level	Feature vector	Sliding window	Example of application
Single site	Certain segment/whole sequence	Yes	Protein interaction site identification
Pairs of sites	Certain segment/whole sequence	Yes	Protein interaction interface identification
Protein	N/A	No	Protein target identification

#### Performance evaluation of site-wise prediction problems

Evaluating the performance of machine learning models is crucial for assessing their predictive accuracy and practical utility. We developed a versatile module that generates a comprehensive set of evaluation metrics, such as precision and recall, specifically for site-wise prediction tasks. This module has been applied to assess the performance of protein–protein interaction site predictions and is adaptable to other site-wise prediction problems, such as identifying disordered residues or assessing the effects of residue variants. It provides a robust framework for evaluating predictive performance across diverse biological contexts.

#### Visualisation

In the current version of PyPropel, we provide simple functions to visually interpret several biological properties. For example, we show the conservation profiles of six example proteins calculated by Jensen-Shannon divergence [30] (see examples in Supplementary Fig. 1). Additionally, the module can be used to plot evaluation metrics for machine learning models and offers flexibility and scalability for both biological property visualization and model performance assessment.

#### Comparison of characteristics with related work

By benchmarking the functionalities of Python-based tools in protein science, we found that most of them work exclusively for feature extraction, especially based on protein sequences rather than protein structures and/or homologous sequences (Table 2). These types of data contain informative features for machine learning [31, 32]. For example, evolutionary information derived from homologous sequences is useful to deduce the conservation of functionally and/or structurally important amino acid sites [33]. In PyPropel, we design modules to systematically extract evolutionary profiles from homologous sequences and generate the features based on protein structures (3di-encoded sequences and states [34] and relative solvent accessibility [35]). In addition, many of the tools hold versatile functionalities for processing DNA and RNA sequences (e.g. PyBioMed [36] and scikit-bio [37]) and a minority of them gain capability to provide end-to-end analysis workflows to allow for the transition from raw data to machine learning applications (e.g. ProPythia [38] and iLearnPlus [9]).

**Table 2:** Comparison of functionalities between different Python-based analysis tools

Tool	Working mode	Sequence type	Statistical analysis / machine learning	MSA analysis	Dataset generation	Feature extraction	Protein structural analysis	Reference
propy	Python inline	Protein	N/A	N/A	N/A	Yes	N/A	[39]
protPy	Python inline	Protein	N/A	N/A	N/A	Yes	N/A	[40]
PyBioMed	Python inline	Protein, DNA, RNA, small molecules	N/A	N/A	N/A	Yes	N/A	[36]
ProteinFlow	Python inline	Protein	N/A	N/A	Yes	Yes	Yes	[41]
peptides.py	Python inline	Protein	N/A	N/A	N/A	Yes	N/A	[42]
ProPythia	Python inline	Protein	Yes	N/A	N/A	Yes	N/A	[38]
scikit-bio	Python inline	Protein, DNA, RNA	Yes	Yes	N/A	Yes	N/A	[37]
PyPropel	Python inline	Protein	N/A	Yes	Yes	Yes	Yes	–
PyFeat	Command line	Protein, DNA, RNA	N/A	N/A	N/A	Yes	N/A	[43]
iFeature	Command line/webserver	Protein	N/A	N/A	N/A	Yes	N/A	[8]
iLearn	webserver	Protein, DNA, RNA	Yes	N/A	N/A	Yes	N/A	[10]
iLearnPlus	webserver	Protein, DNA, RNA	Yes	N/A	N/A	Yes	N/A	[9]

#### Validating the reliability of PyPropel

To gain an understanding of the computational efficiency of PyPropel, we managed to evaluate the runtime of generating three commonly seen features derived purely from protein sequences, including the composition of amino acids (20 dimension), dipeptides (400 dimension), and tripeptides (8000 dimension). Our results demonstrate that PyPropel is among the fastest tools, for example, consuming 1.437 s to generate the tripeptide composition of 10 proteins (Supplementary Fig. 2). In addition, to gain an understanding of the quality of analysis results of PyPropel, we calculate the physical distances of residues (residing in chain A) away from other residues (residing in other chains) in human calcium homeostasis modulators (PDB code: 6UIW). Our results suggest that the interaction interfaces determined based on the distances highly agree with the true landscape of interactions in the native structure (Supplementary Fig. 3). Moreover, to increase the usability of PyPropel, we showcased two end-to-end analysis pipelines in its documentation for predicting interaction sites and drug target interactions, showing the high compatibility of PyPropel with other protein sequence and structural analysis tools (such as TMKit [17] and PyBioMed [36]).

## Conclusion

PyPropel streamlines both the pre- and post-processing of protein sequence data, optimising workflows for bioinformatics and machine learning applications. By integrating functionalities such as automated data retrieval, structural and functional annotation, and the assembly of multisource protein features, PyPropel improves the efficiency of dataset generation and model evaluation. Its ability to seamlessly combine pre-processing, annotation, and feature extraction from diverse tools addresses a gap in current bioinformatics pipelines, providing a comprehensive solution for large-scale protein sequence analysis. The lack of extracting structure-based features is current limitations of PyPropel, which will be addressed for future iterations. As protein data continues to expand, PyPropel offers a valuable resource to accelerate functional research and enhance the characterization of protein sequences, particularly at the sequence- and site-specific levels.

## Availability and requirements

Project name: PyPropel. 

Project home page: https://github.com/2003100127/pypropel

Operating system(s): Windows, macOS, Linux.

Programming language: Python.

Other requirements: Python 3.10 or higher, Numpy 2.0.1 or higher, Pandas 2.2.2 or higher, Seaborn 0.13.2 or higher, Matplotlib 3.9.1 or higher, Biopython 1.84, Scikit-learn 1.5.1.

License: GPL3.0 License.

Any restrictions to use by non-academics: None.

## Supplementary Information

Supplementary 1.

## Data Availability

PyPropel is made freely available at https://github.com/2003100127/pypropel. The usage of PyPropel is shown at https://2003100127.github.io/pypropel. All data used throughout this research can be downloaded at https://github.com/2003100127/pypropel/releases.
