# Supplementary material for: PyPropel: a Python-based tool for efficiently processing and characterising protein data
Source: BMC Bioinformatics. 2025 Mar 1;26:70. doi: 10.1186/s12859-025-06079-3 (PMC11871610; doi:10.1186/s12859-025-06079-3)
Supplement: Supplementary file 1 — Supplementary 1. [file 12859_2025_6079_MOESM1_ESM.docx]

***Supplementary Material***

PyPropel: a Python-based tool for efficiently processing and characterising protein data

Jianfeng Sun^1,#^, Jinlong Ru^2^, Adam P. Cribbs^1^, Dapeng Xiong^3,4,#^

^1^ Botnar Research Centre, University of Oxford, Headington, Oxford OX3 7LD, UK

^2^ Chair of Prevention of Microbial Diseases, School of Life Sciences Weihenstephan, Technical University of Munich, 85354 Freising, Germany

^3^ Department of Computational Biology, Cornell University, Ithaca 14853, USA

^4^ Weill Institute for Cell and Molecular Biology, Cornell University, Ithaca 14853, USA

^#^ Correspondence: [jianfeng.sun@ndorms.ox.ac.uk](mailto:jianfeng.sun@ndorms.ox.ac.uk). [dx38@cornell.edu](mailto:dx38@cornell.edu).


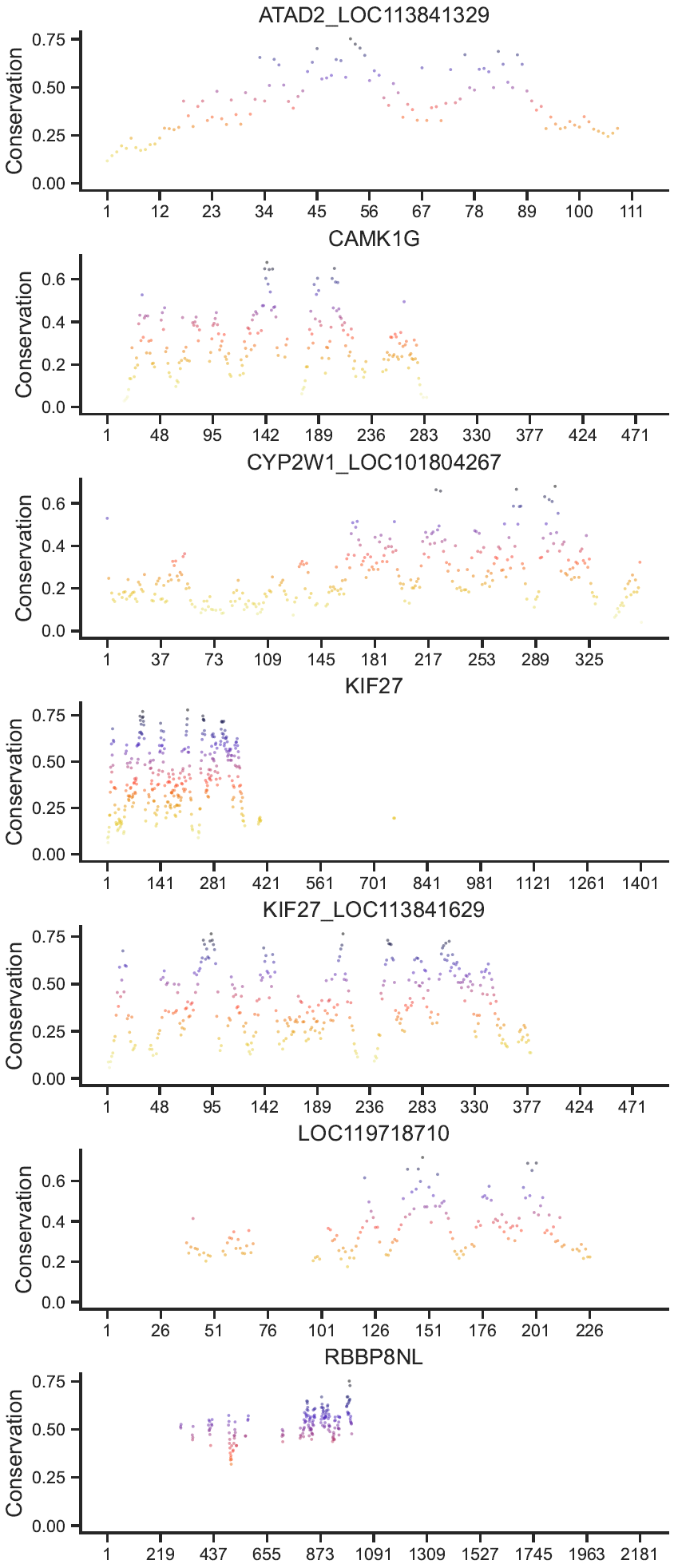


**Figure S1**. Jensen–Shannon (JS) divergence conservation scores of 6 proteins (ATAD2_LOC113841329, CAMK1G, CYP2W1_LOC101804267, KIF27, KIF27_LOC113841629, LOC119718710, and RBBP8NL). The conservation scores are calculated by running the JSD function in PyPropel. This function is a wrapper of the JSD program [1].


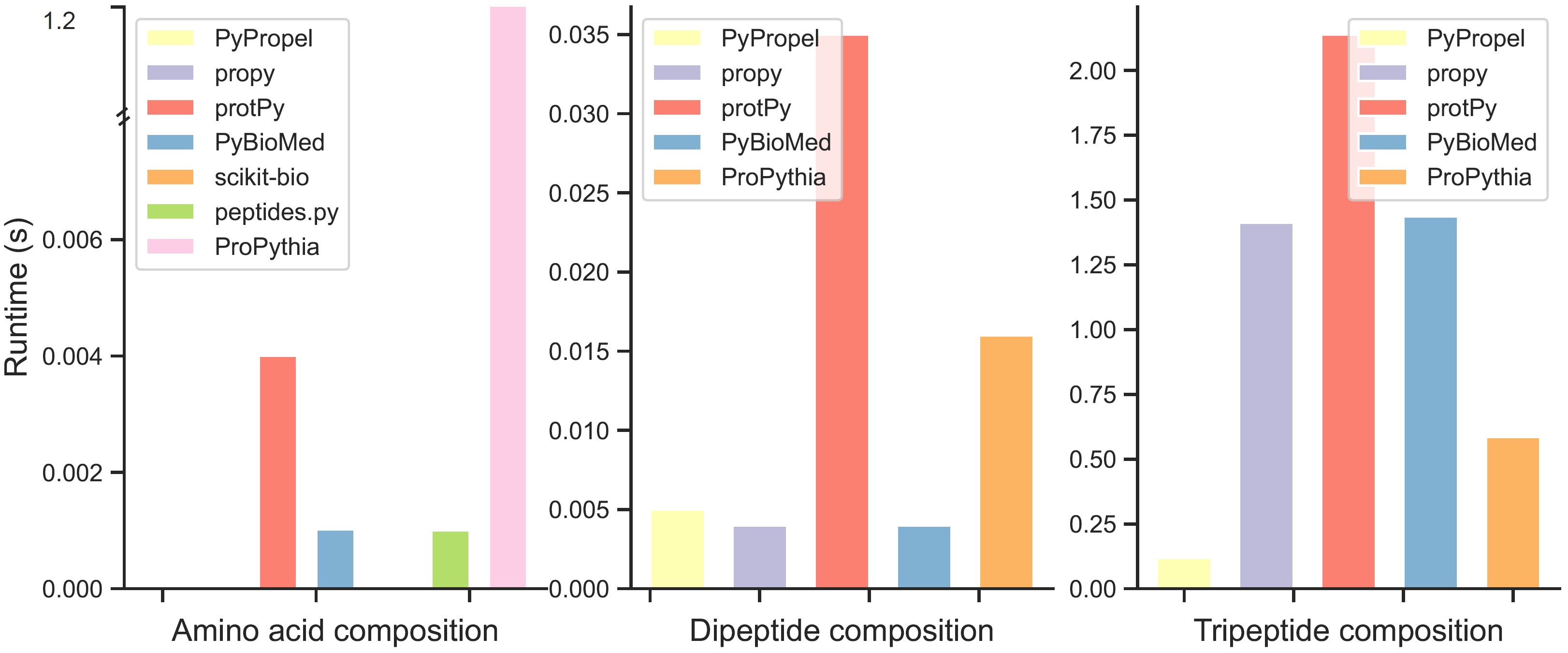


**Figure S2**. Runtime of generating the amino acid composition, the dipeptide composition, and the tripeptide composition by protein analysis tools. The runtime was calculated by competing the three kinds of compositions of 10 protein sequences using each protein analysis tool. The 10 sequences were derived from the ProPythia Github repository [2]. scikit-bio and peptides.py were only evaluated at calculating the amino acid composition due to no functions contained for calculating the dipeptide composition and the tripeptide composition.


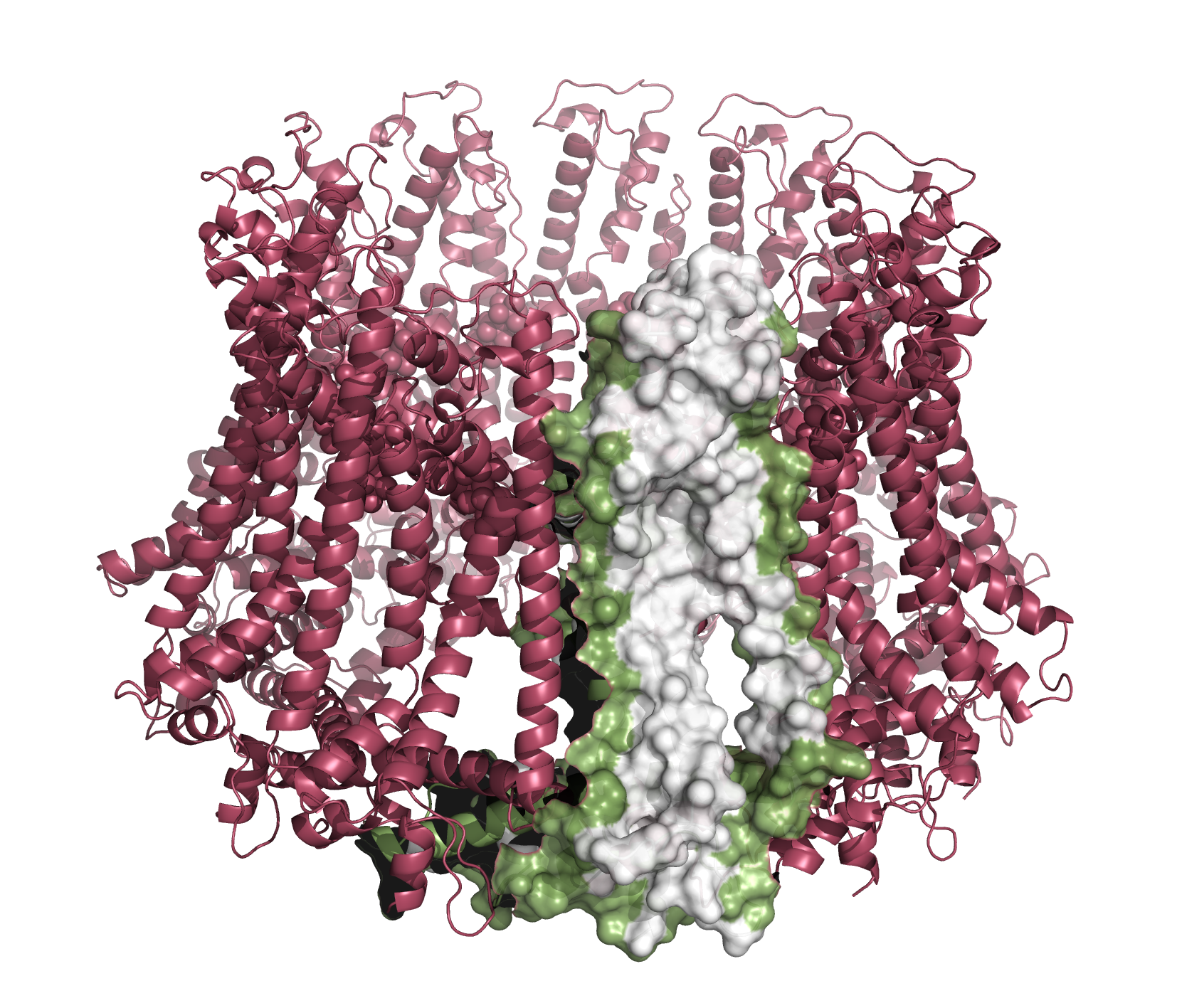


**Figure S3**. Structure of human calcium homeostasis modulators (PDB code: 6UIW) visualised by TMKit and Pymol. The interfaces of chain A (coloured white) interacting with its partners (coloured raspberry) in the protein complex are coloured green. The distances between residues from chain A and those from other chains are calculated using PyPropel. Residues from chain A are seen to be in interaction with residues from other chains if they share less than 5.5 angstrom (Å) (heavy atoms, that is, non-hydrogen [3]).

**Reference**

1. Capra JA, Singh M. Predicting functionally important residues from sequence conservation. Bioinformatics. 2007;23:1875–82.

2. Sequeira AM, Lousa D, Rocha M. ProPythia: A Python package for protein classification based on machine and deep learning. Neurocomputing. 2022;484:172–82.

3. Fuchs A, Martin-Galiano AJ, Kalman M, Fleishman S, Ben-Tal N, Frishman D. Co-evolving residues in membrane proteins. Bioinformatics. 2007;23:3312–9.
